# Supplementary material for: Complexome profiling of the Chlamydomonas psb28 mutant reveals TEF5 as an early PSII assembly factor
Source: Plant Cell. 2025 Mar 17;37(6):koaf055. doi: 10.1093/plcell/koaf055 (PMC12164590; doi:10.1093/plcell/koaf055)
Supplement: koaf055_Supplementary_Data [file koaf055_supplementary_data.zip › psb28_interactive.zip!/plots/intensity/qualityPlots/peptides/DLA2_Cre03.g158900.t1.2.html]

### Cre03.g158900.t1.2

Trivial name: DLA2  
SeqCoverage [median]: 17.60  
Mass: 49.80 kDa  
  
 

| Sequence | MissedCleavage | Mass [Da] | wtIntensity | muIntensity | Score |
| --- | --- | --- | --- | --- | --- |
| AAAPAAAPAK | 0 | 837.47 | 0.00 | 353.11 | 77.06 |
| ATTVSELR | 0 | 875.47 | 0.00 | 1804.22 | 113.50 |
| AVAPAAAPAAAPAAVAAAPAAAPAK | 0 | 2037.14 | 0.00 | 2162.58 | 47.75 |
| GTTKPFSTLQAAVAR | 1 | 1546.85 | 0.00 | 2376.92 | 44.46 |
| ITAADVEAR | 0 | 944.49 | 0.00 | 3328.79 | 103.56 |
| IVATPYAK | 0 | 861.50 | 0.00 | 2187.61 | 60.97 |
| KVMNVNLTADHR | 1 | 1396.72 | 0.00 | 864.63 | 58.75 |
|  |  |  | 0.0 | 13077.9 |  |
